# Propofol Activation of the Nrf2 Pathway Is Associated with Amelioration of Acute Lung Injury in a Rat Liver Transplantation Model

**DOI:** 10.1155/2014/258567

**Published:** 2014-02-11

**Authors:** Weifeng Yao, Gangjian Luo, Guosong Zhu, Xinjin Chi, Ailan Zhang, Zhengyuan Xia, Ziqing Hei

**Affiliations:** ^1^Department of Anesthesiology, The Third Affiliated Hospital of Sun Yat-sen University, No. 600 Tianhe Road, Guangzhou 510630, China; ^2^Department of Anesthesiology, Henan Provincial People's Hospital, People's Hospital of Zhengzhou University, Zhengzhou 450000, China; ^3^Department of Anesthesiology, University of Hong Kong, Hong Kong

## Abstract

*Objective*. This study aimed to investigate whether propofol pretreatment can protect against liver transplantation-induced acute lung injury (ALI) and to explore whether Nrf2 pathway is involved in the protections provided by propofol pretreatment. *Method*. Adult male Sprague-Dawley rats were divided into five groups based on the random number table. Lung pathology was observed by optical microscopy. Lung water content was assessed by wet/dry ratio, and PaO_2_ was detected by blood gas analysis. The contents of H_2_O_2_, MDA, and SOD activity were determined by ELISA method, and the expression of HO-1, NQO1, Keap1, and nuclear Nrf2 was assayed by western blotting. *Results*. Compared with saline-treated model group, both propofol and N-acetylcysteine pretreatment can reduce the acute lung injury caused by orthotopic autologous liver transplantation (OALT), decrease the lung injury scores, lung water content, and H_2_O_2_ and MDA levels, and improve the arterial PaO_2_ and SOD activity. Furthermore, propofol (but not N-acetylcysteine) pretreatment especially in high dose inhibited the expression of Keap1 and induced translocation of Nrf2 into the nucleus to further upregulate the expression of HO-1 and NQO1 downstream. *Conclusion*. Pretreatment with propofol is associated with attenuation of OALT-induced ALI, and the Nrf2 pathway is involved in the antioxidative processes.

## 1. Introduction

ALI occurs after orthotopic liver transplantation (OLT) in approximately 34.2% to 77.8% of cases [1, 2]. Moreover, ALI is one of the main causes of death after OLT [3]. Some studies have suggested that inflammation is the main mechanism and therapeutic target of ALI induced by liver transplantation [4–8]. Recent studies have shown that oxidative stress is another important pathogenic mechanism of ALI [9]. However, the status of oxidative stress in, and effects of antioxidant therapy on ALI induced by liver transplantation are poorly understood.

Propofol (2,6-diisopropylphenol) is one of the most commonly used agents for use in either induction and maintenance of anesthesia or sedation [10]. Apart from its multiple anesthetic advantages, propofol exerts a number of nonanesthetic effects [11]. Propofol has been shown to enhance tissue antioxidant capacity in various tissues in a rat model [12]. Also, recent studies have suggested that propofol takes part in protection of various organs during acute injury. Its cytoprotective properties might be related to antioxidative stress [13] and anti-inflammatory response [14]. HO-1 has been reported to be involved in the antioxidative mechanism of propofol [15–18].

Nrf2 is a member of the Cap “n” Collar (CNC) family of basic leucine zipper (bZip) transcription factors which includes NF-E2, Bach1-2, and Nrf1-3 [19]. Nrf2 is a stress-sensing genetic transcription factor which appears to be a master regulator of cellular responses to oxidative damage and other stressful conditions. The Nrf2 antioxidant response pathway is “the primary cellular defense against the cytotoxic effects of oxidative stress” [20]. The transcriptional activity and protein stability of Nrf2 are principally regulated by a BTB-Kelch protein, Keap1, which has been shown to be a substrate adaptor protein for the Keap1-cullin (Cul)3-Rbx1 E3 ubiquitin ligase complex, which, in turn, is responsible for Nrf2 degradation [21]. Keap1 targets Nrf2 for ubiquitination and subsequent degradation by proteasome. Like other members of the CNC family of bZip transcription factors, Nrf2 forms a heterodimer with its obligatory partner Maf, thereby binding to a *cis*-acting enhancer sequence known as the antioxidant response element (ARE). This regulates the basal and inducible expression of genes that can be grouped into several categories which include phase II detoxifying enzymes, antioxidant genes, scavenger receptor, chaperone proteins, transcription factors, and transporters [22]. Furthermore, among the antioxidant and detoxification enzymes, HO-1 is the most important one [23, 24]. Recent studies have shown that activation of Nrf2 plays an important role in acute lung injury both in vitro and in vivo [25, 26]. Nonetheless, Nrf2 antioxidant response in the process during OALT-induced ALI remains unknown.

The aim of the current study was to determine whether propofol could attenuate ALI, and if so, whether activation of Nrf2 antioxidant response pathway was involved in a rat model of liver transplantation “http://www.ncbi.nlm.nih.gov/pubmed/24116032”.

## 2. Methods and Materials

### 2.1. Animals and Establishment of the OALT Model

This study was approved by the Animal Care Committee of the Sun Yat-sen University and performed in accordance with the National Institutes of Health guidelines for the use of experimental animals [27]. A total of 40 pathogen-free, adult male Sprague-Dawley rats (weighing 200–250 g) were housed in individual cages in a temperature-controlled room with alternating 12 h light/dark cycles. The rats were acclimated for one week prior to experiments. Food was withheld 8 h before the start of experiments, but all animals had free access to water. Rats were anesthetized with pentobarbital 30 mg/kg body weight, i.p. under 50% oxygen delivered by animal mask. A standard model of OALT was created as previously described [28]. The model simulates the main pathophysiological processes during perioperative period of liver transplantation. The anhepatic phase can be controlled without the development of the complications of rejection or other nonsurgical factors in orthotopic liver transplantation using the cuff technique.

### 2.2. Animal Groups

The experimental animals were randomly divided into five groups of eight rats each: sham: celiotomy and vascular separation without OALT, treated with saline (S), model (M), low-dose propofol (LP), high-dose propofol (HP), and NAC groups (NAC). Groups S and M were injected intraperitoneally in 3 mL normal saline. for 3 consecutive days. Groups LP, HP, and NAC were injected with propofol (Diprivan, propofol 1%, CG411, AstraZeneca, Caponago, Italy. 50 mg/kg, i.p. or propofol 100 mg/kg, i.p.) [29], and NAC (W508454. Sigma Aldrich (UK), 150 mg/kg, i.p.) [30] for three consecutive days. The last injection of propofol occurred 12 h prior to operation. The rats in the Group S were subjected to only celiotomy and vascular separation under anesthesia. The rats in other groups were subjected to OALT operation, including hepatic vascular occlusion for 20 ± 1 min.

## 3. Tissue Sampling

The animals were anesthetized with chloral hydrate at 8 h after OALT. The thorax was opened and lungs were removed. The middle lobes of the right lung were weighed on an electronic scale, and the inferior lobes of right lung tissue were fixed in 10% buffered formalin and embedded in paraffin for histologic evaluation. The rest of the lung tissues were quickly transferred in liquid nitrogen for storage for protein assays, and for measuring H_2_O_2_ and malondialdehyde (MDA) levels and SOD activity.

### 3.1. Arterial Blood Gas Analysis

The arterial blood partial pressure of oxygen (PaO_2_) was measured in each lung sample using an iSTAT portable blood gas analyzer (Abbott Laboratories, USA) according to the manufacturer's instructions.

### 3.2. Lung Histologic Evaluation

The lung tissues were sectioned (about 4 mm thick) and stained with hematoxylin and eosin (H&E). Two pathologists who were blinded to the protocol analyzed the samples and scored the pathology as described by Derks and Jacobovitz Derks [31]. The grade of edema of the alveolar mesenchyme, intra-alveolar cell infiltration, and alveolar hemorrhage was also scored.

### 3.3. Water Content of Lung

The wet weights of the superior lobes of the right lung were measured, and the samples heated at 80°C for 24 h to reach a constant weight. The water content of the lung was calculated: water content = (lung wet weight − lung dry weight)/lung wet weight × 100 [32].

### 3.4. Western Blot Analysis

The Nrf2, Keap1, HO-1, and NQO1 proteins were detected by western blot analysis. The lung tissues were finely homogenized, suspended in ice-cold lysis buffer (1.5 mL/g of tissue), and then centrifuged at 12,000 g for 10 min at 4°C. The supernatants were recovered for subsequent analysis. After measuring the protein concentrations of each sample, a 50 *μ*g of the protein sample was solubilized in sodium dodecyl sulfate (SDS) loading buffer. The samples were loaded into a 10% SDS polyacrylamide gel, electrophoresed, and then transferred to a polyvinylidene difluoride (PVDF) membranes. Finally, the PVDF membranes were incubated with Nrf2 antibodies (1 : 250, sc-722, Santa Cruze), Keap1 antibodies (1 : 1000, ABS97, Millipore), HO-1 (1 : 250, sc-10789, Santa Cruz), or NQO1 (1 : 250, sc-32793, Santa Cruz) followed by the corresponding secondary antibodies. Protein-antibody complexes were detected with an enhanced chemiluminescence system (KGP1125, purchased from Nanjing KeyGen Biotech. Co., Ltd.). Protein band sizes were estimated using AlphaView software (Cell Biosciences, Santa Clara, CA, USA). Anti-Histone H2A (1 : 500, Santa Cruz, USA) and anti-*β*-actin (1 : 1500, Merck Milipore, Germany) were used to detect proteins levels. The density measurement was correlated to the protein levels and normalized to those of *β*-actin.

### 3.5. Detection of H_2_O_2_ and MDA Levels and SOD Activity

Lung tissues were prepared as 10% tissue homogenates, and centrifuged at 3000 rpm at 4°C for 10 min. The supernatant was collected for further analysis. The levels of H_2_O_2_ were measured using a kit (Keygen Biotech. Co., Ltd., Nanjing, China). The MDA content was detected according to the instructions of the MDA kit purchased from the Nanjing Jiancheng Bioengineering Institute (Nanjing, China). The SOD activity was detected according to the instructions of the SOD Assay Kit (Keygen Biotech. Co., Ltd., Nanjing, China).

### 3.6. Statistical Analysis

All data were described as means ± SD and analyzed using SPSS 13.0 software (SPSS, Chicago, IL, USA). The differences between groups were analyzed by one-way, ANOVA. Multiple comparisons were performed by the Bonferroni procedure. Correlation between different variables was assessed by Spearman's coefficient. Differences with *P* < 0.05 were considered statistically significant.

## 4. Results

### 4.1. Light Microscopy of Lung Histology

**Figure 1:**
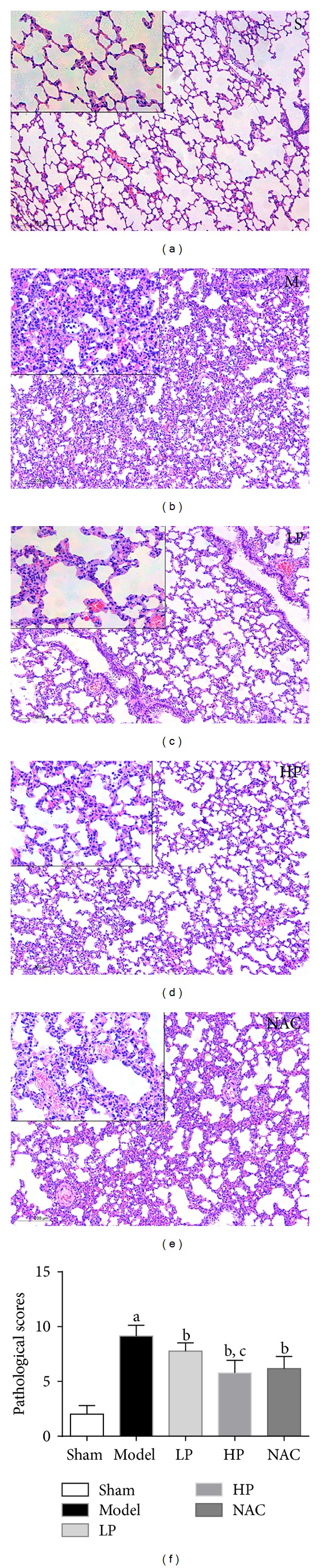
Pathological changes in the lungs after OALT. Group S: sham-operated group; Group M: saline-treated model group; Group LP: low-dose propofol intervention group; Group HP: high-dose propofol intervention group. H&E staining; ×100. Bar graph quantifies the lung histological scores. Data are presented as mean ± SD, with *n* = 8 per group. (a)  *P* < 0.05 versus Group S; (b)  *P* < 0.05 versus Group M; (c) *P* < 0.05 versus Group LP.

Pathological morphology of the lung following orthotopic autologous liver transplantation was assessed using H&E staining. Lung from the sham group (Group S) had normal morphology, with minimal inflammatory cell infiltration (Figure 1(a)). In Group M, there was infiltration of polymorphonuclear and mononuclear inflammatory cells into the intra-alveolar and interstitial spaces. This phenomenon was accompanied by interstitial edema, and pulmonary architectural damage (Figure 1(b)). However the ALI induced by OALT was significantly attenuated in rats pretreated with propofol, especially at high doses and NAC (Figures 1(c)–1(e)), as shown by the histopathological scores (Figure 1(f)).

### 4.2. Water Content and PaO_2_ of Lungs

**Figure 2:**
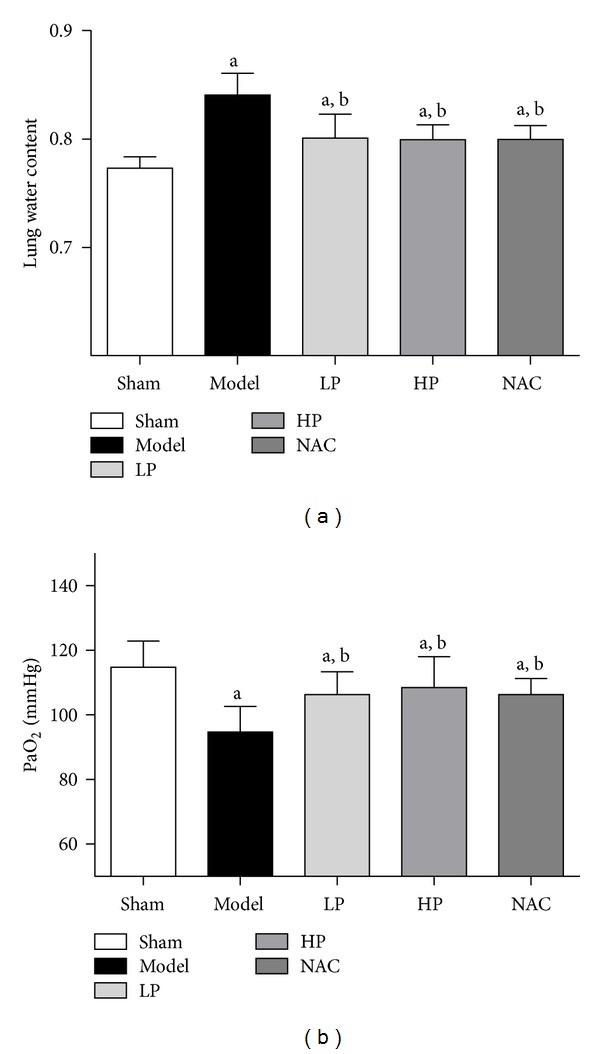
Lung tissue water content (Figure 2(a)) and PaO_2_ (Figure 2(b)) after OALT. Group S: sham-operated group; Group M: normal saline control model group; Group LP: low-dose propofol intervention group; Group HP: high-dose propofol intervention group. Data are presented as mean ± SD.(a)  *P* < 0.05 versus Group S; (b)  *P* < 0.05 versus Group M.

To quantitatively assess the extent of pulmonary edema after OALT, the water content of the middle lobe of the right lung was measured. The lung water content was significantly higher in rats that received OALT (Groups M, LP, HP, and NAC, *P* < 0.05, versus Group S), whereas the water content was lower in rats pretreated with propofol or NAC compared with Group M (Figure 2(a)). PaO_2_ was measured to evaluate pulmonary blood gas barrier dysfunction. PaO_2_ was significantly higher in Groups M, LP, HP, and NAC, *P* < 0.05, versus Group S, and lower in rats pretreated with propofol or NAC compared with Group M (Figure 2(b)).

### 4.3. H_2_O_2_ Levels, SOD Activity and MDA Levels in Lung Tissue and Pathological Scores

**Figure 3:**
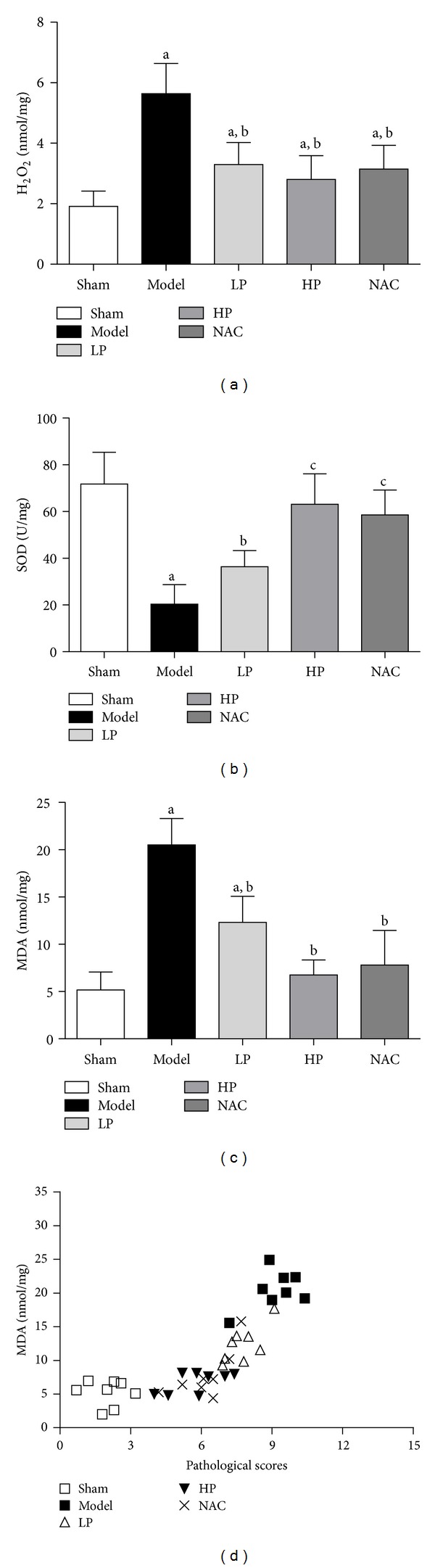
H_2_O_2_ levels, SOD activity, and MDA levels in lung tissue after OALT ((a), (b), and (c)). Correlations between MDA levels and pathological scores (d). Group S: sham-operated group; Group M: normal saline control model group; Group LP: low-dose propofol intervention group; Group HP: high-dose propofol intervention group. Data are presented as mean ± SD. (a)  *P* < 0.05 versus Group S; (b)  *P* < 0.05 versus Group M; (c)  *P* < 0.05 versus Group LP.

H_2_O_2_ is one of the main reactive oxygen species (ROS) which can induce lipid peroxidation. Malondialdehyde (MDA) is the end product of lipid peroxidation and can indirectly reflect the extent of the radical attack on cells. Superoxide dismutase (SOD) can protect cells from damage by elimination of oxygen free radicals. It plays an important role in maintaining the balance between oxidation and antioxidation. As shown in Figure 3, the levels of H_2_O_2_ and MDA were significantly higher in rats that received OALT compared with those in the sham group (Groups M, LP, HP, and NAC, *P* < 0.05, versus Group S). This was accompanied by lower SOD activity. However, the levels of H_2_O_2_ and MDA were lower in rats pretreated with propofol (especially high dose) or NAC compared with Group M. This was accompanied by higher SOD activity. Studies have shown that NAC can significantly increase the activity of SOD and scavenge ROS [33]. Also, MDA is a powerful biomarker of oxidative stress in an organism. Its levels were positively correlated with lung pathological scores (*r* = 0.7993; *P* < 0.001), which indicates the important role of oxidative stress in the pathophysiologic process of ALI induced by OALT. All the above results suggest that propofol inhibits lung oxidative stress after OALT in a dose-dependent manner.

### 4.4. Keap1 and Nuclear Nrf2 Expression in Lung Tissue

**Figure 4:**
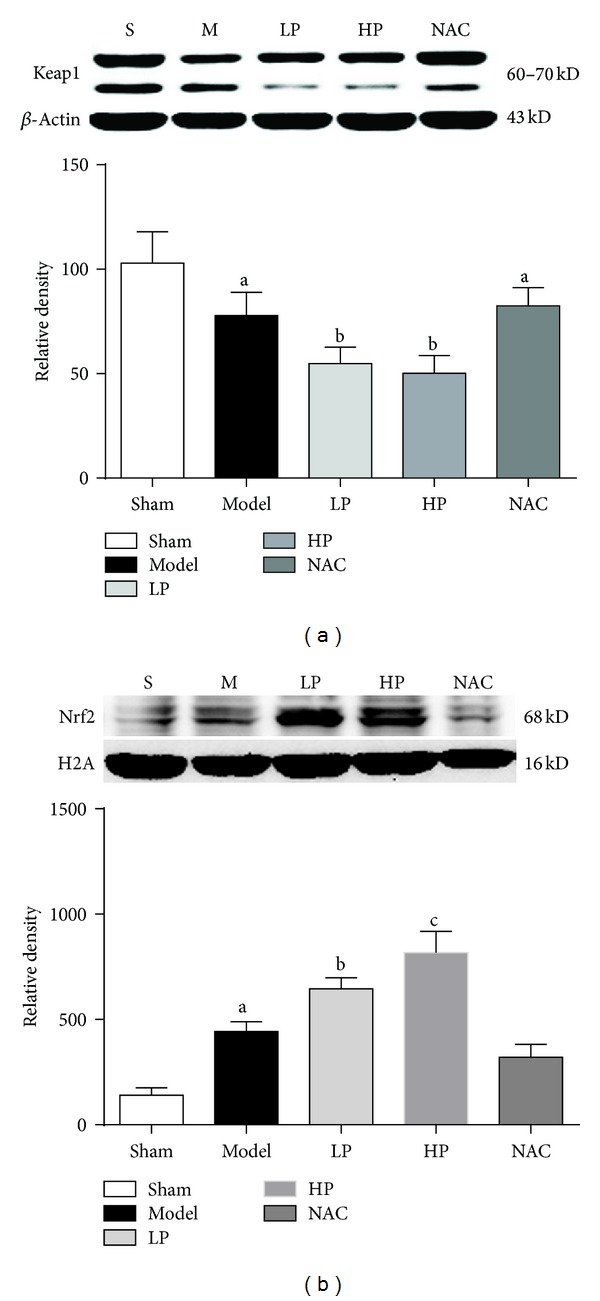
Keap1 and nuclear Nrf2 expression in lung tissue after OALT. Group S: sham-operated group; Group M: normal saline control model group; Group LP: low-dose of propofol intervention group; Group HP: high-dose propofol intervention group. Data are presented as mean ± SD. (a)  *P* < 0.05 versus Group S; (b)  *P* < 0.05 versus Group M; (c)  *P* < 0.05 versus Group LP.

As shown in Figure 4, the expression levels of Keap1 were lower in rats that received OALT (Groups M, LP, HP, and NAC, *P* < 0.05 versus Group S). The expression levels of Keap1 were significantly lower in rats pretreated with propofol compared with those in Group M (Group LP and HP; *P* < 0.05 versus Group M). The levels of nuclear Nrf2 was significantly higher in rats that received OALT (Groups M, LP, HP, and NAC, *P* < 0.05, versus Group S), and the expression was significantly higher in rats pretreated with propofol compared with those in Group M (Groups LP and HP, *P* < 0.05, versus Group M). However, the levels of Keap1 and nuclear Nrf2 in Group NAC were not significantly different compared with those of Group M.

### 4.5. HO-1 and NQO1 Expression in Lung Tissue

**Figure 5:**
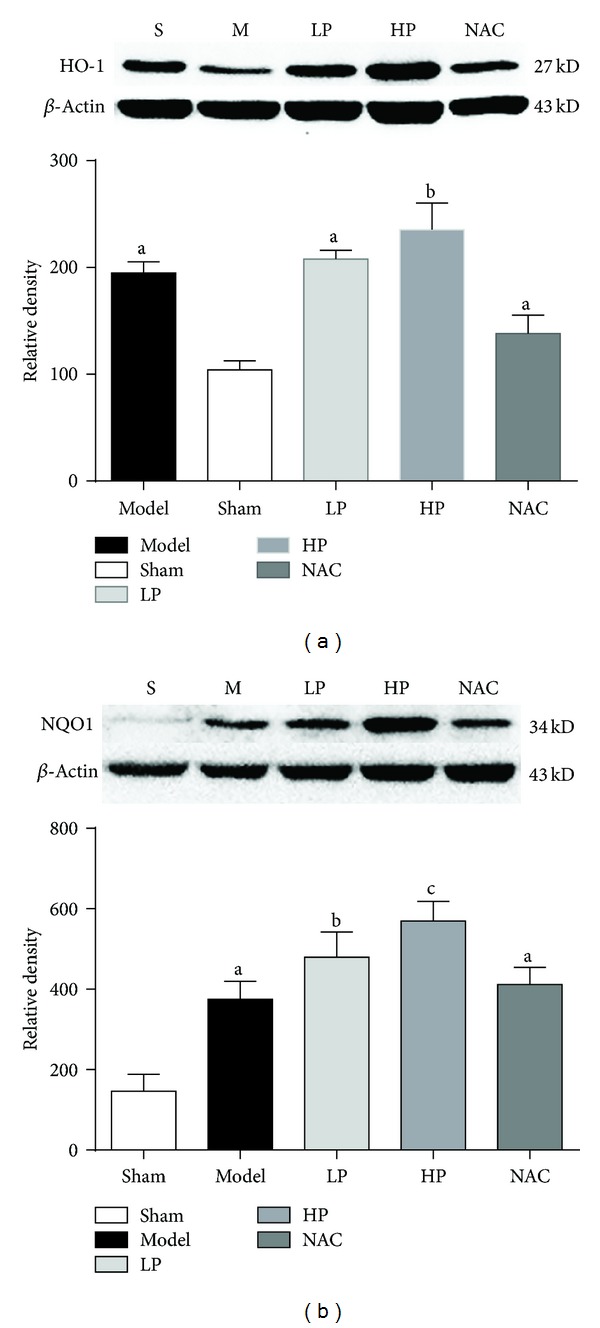
HO-1 and NQO1 levels in lung tissue after OALT. Group S: sham-operated group; Group M: normal saline control model group; Group LP: low-dose of propofol intervention group; Group HP: high-dose propofol intervention group. Data are presented as mean ± SD. (a)  *P* < 0.05 versus Group S; (b)  *P* < 0.05 versus Group M; (c)  *P* < 0.05 versus Group LP.

HO-1 and NQO1 are regulated downstream by Nrf2. Measurement of their expression can reflect the capacity of Nrf2 to regulate antioxidants. As shown in Figure 5, the levels of HO-1 and NQO1 were significantly higher in rats that received OALT (Groups M, LP, HP, and NAC, *P* < 0.05, versus Group S), especially in rats pretreated with propofol (Groups LP and HP, *P* < 0.05, versus Group M).

## 5. Discussion

**Figure 6:**
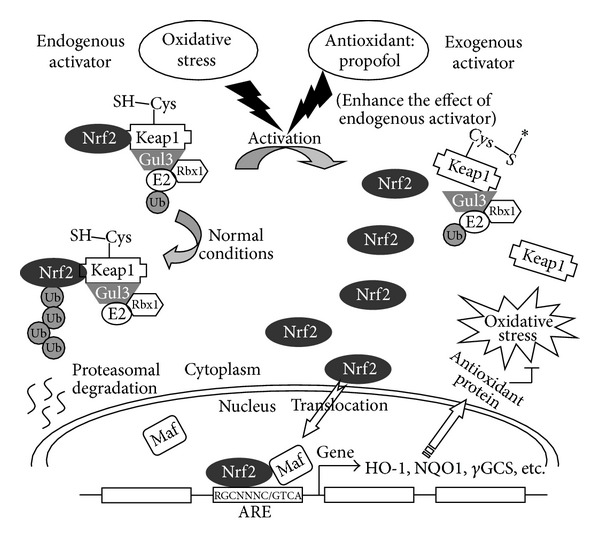
A diagram of propofol-induced activation of Nrf2 under conditions of oxidative stress. Under normal conditions, a single Keap1 protein is able to target multiple Nrf2 proteins for destruction by the ubiquitin-proteasome system. Endogenously generated ROS alter the interaction between Nrf2 and its repressor under oxidative stress, resulting in the accumulation of Nrf2 in the cytoplasm and Nrf2 translocates to the nucleus. As an antioxidant, propofol enhances the effect of the endogenous activator of Nrf2 pathway. Propofol accelerates the dissociation of Nrf2 from Keap1 which leads to more Nrf2 translocation to the nucleus under conditions of oxidative stress. Through binding with Maf and ARE, Nrf2 regulates the expression of its downstream target genes (HO-1, NQO1, *γ*-GCS, etc.) to prevent oxidative stress and damage.

The main findings of the present experiments were that propofol reduced the decline in oxygenation, pulmonary edema (as assessed by increase in the W/D ratio), reactive oxygen species (H_2_O_2_) and lipid peroxidation (MDA), and histologic alteration induced by OALT through the Nrf2 antioxidant response pathway. To the best of our knowledge, this is the first report showing that the antioxidant pathway mediated by Nrf2 is associated with protective effect of propofol (Figure 6) in ALI induced by liver transplantation.

Lung injury and respiratory failure often herald the development of multiorgan dysfunction after liver transplantation and other major surgeries. Systemic generation of ROS during injurious insults impairs the pulmonary endothelium, initiating deterioration in oxygenation [34]. Specifically, we have previously shown that inflammation during OALT increased alveolocapillary permeability in the lung [35], and Nrf2 played an important role in antioxidative stress during OALT [36]. Based on these results, we investigated propofol as a preventive agent because of its potent antioxidative stress ability. Indeed, in the present study, the extent of the increase of H_2_O_2_ and MDA in the rabbits that underwent OALT were reduced by propofol while SOD, HO-1, and NQO1 were elevated. This result is similar to that reported by Vasileiou et al. [37], who found that propofol could efficiently prevent IIR-induced lung injury by reducing antioxidative stress. The results are in contrast to the report by Kwak et al. which demonstrated that propofol attenuates endotoxin-induced ALI in rabbits mainly by inhibiting neutrophil and IL-8 responses in the inflammatory responses [38].

Nrf2 is ubiquitously expressed in various organs including lung [39]. A previous study suggested that activation of Nrf2 is involved in the prevention against ALI induced by radiation and hyperoxia [40, 41]. And most studies have documented that Nrf2 is a key regulatory transcription factor for inducing antioxidant and detoxification genes [42]. Upregulation of Nrf2 can promote the synthesis of various antioxidant enzymes that protect against the adverse effects of oxidative stress induced by ROS [43]. Conversely, downregulation of Nrf2 makes the organism more sensitive to oxidative stress induced by ROS [44]. Using in vivo study, Nrf2 knockout mice were found to be more susceptible to injury and tumorigenesis in response to oxidative and chemical stress [45]. Nrf2 is activated following its detachment from Keap1 and then translocated to the nucleus where it binds to the ARE in the promoter region of target genes, leading to their transcriptional induction. Endogenous antioxidants are activated, and ROS are scavenged to reduce oxidative damage [43]. There are more than 200 genes that have been shown to be regulated by the Nrf2/ARE pathway. These genes encode phase II detoxification enzymes and antioxidant proteins, including NQO1 and HO-1 [42]. HO-1 is an enzyme that catalyzes the breakdown of heme into the antioxidant biliverdin, the anti-inflammatory agent carbon monoxide, and iron [46]. NQO1 is an enzyme that, in humans, is encoded by the NQO1 gene. The enzymatic activity of this protein prevents the one-electron reduction of quinones that results in the production of free radical species [47]. In the present study, Nrf2 antioxidant response pathway was activated by oxidative stress during OALT-induced ALI. However, the levels of Nrf2, HO-1, and NQO1 apparently were insufficient to prevent lung oxidative damage induced by OALT, as serious lung injury still occur. These results indicate that enhanced antioxidant activity mediated by Nrf2 can reduce oxidative injury.

Mounting evidence has shown that propofol can reduce remote organ ischemia/reperfusion injury through its antioxidant effect [37, 48]. However, the mechanisms of propofol effects on various antioxidants remain to be identified. The protective role of propofol is probably mostly related to its preischemic administration, since postischemic administration has not been found effective [49]. This could be either due to adequate concentrations of propofol locally in the lung tissue and systemic circulation at the onset of reperfusion or also due to a protective effect during ischemia. In order to prove the influence of propofol on the Nrf2 pathway, we chose another antioxidant NAC as control. The effect of ALI induced by OALT in the presence and absence of NAC on nuclear Nrf2 was evaluated, with inhibition of this process by NAC taken as evidence that the process was redox mediated. We found that propofol efficiently enhanced the activities of antioxidant enzymes mediated by Nrf2, which was characterized by the downregulation of Keap1 expression and upregulation of nuclear Nrf2, HO-1, and NQO1 expression. Also, the activity of SOD was increased and oxidative damage in injured lung was significantly reduced when rats were pretreated with propofol especially in high dose. Interestingly, we also found that NAC could increase SOD activity but cannot activate Nrf2 pathway, which suggested that NAC played a role antioxidant mainly through improving activity of SOD rather than activating Nrf2 pathway. This result is similar to the report by Messier et al. who suggested that the NAC cytoprotective effect is independent of nuclear Nrf2 [50]. Taken together, our results suggest that propofol is a potential Nrf2 strong activator.

There are some limitations in this study. Although we found that propofol activation of the Nrf2 pathway prevents OALT-induced ALI, we did not investigate whether propofol could still be protective in the presence of downregulation of Nrf2 expression. Such studies are planned in the future. Future studies will be directed at the activation and roles of Nrf2 pathways after propofol preconditioning of the lung. Treatment initiated after the onset of ischemia or during reperfusion due to unexpected occurrence of I/R event is also an area of proposed future study.

In conclusion, we found that Nrf2-mediated defense mechanisms are involved in the antioxidant ability of propofol against the adverse effects of acute lung oxidative injury induced by OALT. In the future, specific target sites of propofol involvement in the Nrf2 pathway, and optimization of treatment for ALI induced by liver transplantation will be studied.
